# Editorial: Health literacy and disease prevention, volume II

**DOI:** 10.3389/fpubh.2024.1369146

**Published:** 2024-02-27

**Authors:** Ozden Gokdemir, Poonam Kushwaha, Deep Shikha, Ferdinando Petrazzuoli, Sudip Bhattacharya

**Affiliations:** ^1^Department of Community Medicine, Izmir University of Economics, Izmir, Türkiye; ^2^Department of Community Medicine, Rama Medical College Hospital and Research Centre, Kanpur, Uttar Pradesh, India; ^3^Himalayan Institute of Medical Sciences, Swami Rama Himalayan University, Dehradun, India; ^4^Center for Primary Health Care Research, Lund University, Malmö, Sweden; ^5^Department of Community Medicine, All India Institute of Medical Sciences, Deoghar, Jharkhand, India

**Keywords:** editorial, health literacy, disease prevention and control, health promotion, public health

## Introduction

In the twenty-first century, societies driven by knowledge face a dilemma in making health-related decisions. Individuals are ill-prepared and lack support in addressing the increasing challenges of navigating complex healthcare systems and making choices for personal and family wellbeing (1). Even those with higher education find it challenging to navigate healthcare systems in so-called “modern” societies, and education systems often fall short of providing people with the necessary skills to access, comprehend, evaluate, and apply information for improving their health. The quality and quantity of available evidence are influenced by factors such as the field, environment, healthcare system, or methodologies employed. Low health literacy levels have a significant negative impact on health placing a significant burden on the financial and personnel resources of the health system of any country (2). Policy initiatives to increase the health literacy epidemic are challenging due to multiple reasons. This editorial aims to contribute to reversing this situation by advocating health literacy by addressing micro, meso, and macro-level factors (3).

The Health Literacy and Disease Prevention series was launched 3 years ago. Its second volume included 22 research articles contributed by 138 authors and many reviewers around the globe. The series was launched with the goals of compiling the best available data on the subject, derived from complex scientific research and reviews and identifying policy implications and specific actions to translate these concepts into tangible results.

### Examples from Europe

The World Health Organization (WHO) has released the survey titled “Conceptual Model of Health Literacy of the European Health Literacy Survey,” where eight European countries contributed sample sizes 1,000 for validating this questionnaire. This dataset is the determinant of four levels of HL: which are “inadequate, problematic, sufficient, and outstanding.” Limited HL was operationalized as either poor or problematic HL, aiming to identify populations facing vulnerabilities. These statistics highlighted significant disparities and facilitated comparisons within and across these nations (4).

Higher levels of health literacy were associated with improved educational attainment, better self-reported overall health, and elevated indices of empowerment. Healthcare providers must acknowledge that people have varying degrees of HL, particularly those who have less education and self-reported health issues. Customizing health information to a person's HL level is essential to encouraging proactive disease management and giving people the confidence to take charge of their health (5).

Being health literate is essential to be able to get enough health information, navigate the healthcare system, receive the correct care, and be able to take charge of your health. As such, it is a significant predictor of health. According to Masquillier et al., innovative methods are needed to improve HL among socioeconomically disadvantaged people. Published research indicates that this innovation needs to offer dependable and understandable health information that is specific to the target population, facilitate “low-threshold access” to health resources through outreach in the community, and help people feel confident enough to act on that information. To meet this requirement, this article describes how the Integrated Community Care (ICC) framework was developed and put into use.

### Examples from Asia

Abu-Humaidan et al., demonstrated satisfactory knowledge, attitudes, and practices (KAP) toward TB among Jordanian university students. The study emphasized the importance of raising awareness of TB transmission routes, treatment options, and availability, particularly among students pursuing careers unrelated to healthcare. It also revealed positive health-seeking behavior about tuberculosis, but it also revealed a concerning tendency of university students to question the value of mask wear. It makes sense to investigate the KAP toward tuberculosis among Jordanian refugee populations and risk groups to reduce the burden of disease going forward.

A study was conducted by Al-Gburi et al., on sexually transmitted infections among Baghdad University students. They reported that most participants had moderate to high knowledge, but there were gaps in knowledge regarding systemic symptoms of STIs and HIV. Respondents agreed on the need for sex education, but traditional and religious barriers were cited as major obstacles. There are specific knowledge gaps for certain high-risk groups, and negative attitudes and stigma should be addressed by increasing focused STI knowledge.

Li et al., conducted a study to analyze health literacy trends in China from 2008 to 2020 and found an improvement from 6.48 to 23.15%. However, significant disparities were noted between urban and rural areas, and the Eastern region had higher health literacy than the Central and Western regions. Age, education level, and social development index were found to be correlated with health literacy.

Marzo, ElSherif et al., examined the factors affecting burnout, resilience, and quality of life (QoL) among healthcare workers (HCWs) during the COVID-19 pandemic in Malaysia. Nurses were more susceptible to burnout and had lower resilience than doctors but reported higher QoL. Older HCWs had higher resilience, while younger and less experienced HCWs were overburdened. Higher-income was associated with better resilience, while longer work hours led to lower QoL. Policymakers and healthcare practitioners must prioritize interventions that promote a healthy workplace environment, address ethical concerns, and prevent burnout among HCWs. Managing long working hours could improve resilience, burnout, and QoL among HCWs.

In Malaysia, Marzo, Khaled et al., explored factors influencing parents' hesitancy to vaccinate their children. Based on their findings, they found that parents with higher levels of education tend to be more doubtful and believe that the vaccine is harmful and inefficient for their kids. It is critical to disseminate the required information about vaccine safety to the educated group.

Tao et al., examined the relationship between Ningbo citizens' knowledge of COVID-19 and HL. The knowledge of COVID-19 is associated with the following factors: age, gender, marital status, education, occupation, annual household income, and chronic conditions. To improve individuals' health literacy and stop the development of COVID-19, different demographic groups should undertake targeted health education and promotion programs.

To create a robust health literacy assessment in Hong Kong, Tian et al., conducted a study to develop the Hong Kong Health Literacy Scale (HLS-HK) and assess its “psychometric properties” among the Chinese adult population. The study concludes that the HLS-HK serves as a valid and dependable tool, and offers valuable insights into the challenges individuals might encounter when utilizing health-related information and services.

Wilandika et al., conducted a systematic review to describe the roles of nurses in promoting patient HL and identify its determinants. By facilitating patients' access to, comprehension, evaluation, and use of health information linked to their conditions, nurses can help patients become more health literate. They also need to be aware of the several elements that affect health literacy and take advantage of these elements to maximize the advancement of health literacy. Nurses can tackle health issues and enhance patient care by using a health literacy approach.

A study by Yang et al. found that the average rate of knowledge of cancer prevention and control measures among adults in Fujian Province was below the 70% target set by the Chinese Department of Health. Those living in urban areas, with white-collar jobs, married, having a bachelor's degree, family history of cancer, and good health, had higher knowledge. These findings can help policymakers design interventions to improve the general population's understanding of cancer prevention and control.

Correct medicine dosing is a crucial component of the safe and effective delivery of oral liquid medicines, particularly for the pediatric population. Younas et al., conducted a pre- and post-intervention survey to assess the knowledge and practice of university students for the same. The use of a tablespoon was significantly reduced, low-volume spoons were preferred, and a wide range of home spoons were rejected. The post-intervention survey also revealed a significant improvement in the proper naming of spoons, the definition of the acronym “tsp,” and the accurate amount of a standard teaspoon. They promoted the use of straightforward instruments, such as awareness seminars and brief video presentations, to enhance the appropriate administration and selection of dosing aids for oral liquid dose forms. This would reduce medication safety concerns and treatment failures.

### Examples from Africa

Authors have created HL resources for Africa, but there is still a shortage of research. Research in 14 African nations showed low HL rates in rural, hard-to-reach areas and difficulties in understanding questionnaire responses. HL assessment tools specific to Africa are necessary (6).

Sah et al., opined that stigma about monkeypox in healthcare is a significant issue. Healthcare workers may conceal their condition due to stigma, posing a threat to transmit the infection. Educational campaigns to reduce stigma and urge infected cases to report their infection are necessary. Culturally acceptable health-promoting messages that provide accurate and timely information on monkeypox are essential. This helps create an environment where the disease and its effects can be discussed openly, honestly, and effectively, helping to prevent and control the disease and the outbreak.

### Examples from America

Improving health literacy in Latin America requires a robust approach. International organizations recommend concerted efforts across sectors to enhance health literacy, improve outcomes, and reduce costs. Despite awareness among government officials, the issue remains inadequately addressed. There is a lack of understanding about how to achieve the goal of increasing health literacy (7).

### Examples from Australia

Australia and other countries have policies for public health. Health literacy is poor in 60% of adult Australians, which affects their ability to make informed healthcare decisions. People with poor health literacy use more healthcare services and have worse health outcomes (8).

To ensure authentic engagement of all stakeholders and generate contextually appropriate, culturally sensitive, and implementable multisectoral solutions to identify the HL strengths, needs, and preferences of the former refugee community in Melbourne, Australia, Jawahar et al., presented a protocol that is a detailed adaptation of the Ophelia (Optimizing Health Literacy and Access) process. This protocol will develop and test new and improved approaches that are likely to be useful for community-based organizations and health services to thoroughly investigate and improve communication, services, and outcomes among disadvantaged groups, particularly migrants and refugees.

## Conclusion

This Research Topic emphasized that HL is determined by various factors as mentioned above. To improve KAP, certain culturally acceptable, target-specific, health-promoting messages as well as required information about the epidemiology of the disease, its prevention, and treatment as well as other health-related practices should be disseminated through the Integrated Community Care approach. It also advocates the provision of a healthy workplace environment, addressing ethical concerns of HCWs, to improve resilience, burnout, and QoL so that they can tackle health-related issues and enhance patient care. This can be done by only developing and adapting a valid and dependable HL tool. Utilizing straightforward digital instruments, such as awareness seminars and brief video presentations can enhance HL, by enabling individuals to make informed choices.

## Author contributions

OG: Writing—original draft, Writing—review & editing. PK: Writing—original draft, Writing—review & editing. DS: Writing—original draft, Writing—review & editing. FP: Writing—original draft, Writing—review & editing. SB: Writing—original draft, Writing—review & editing.
